# Corporate Reporting on Farm Animal Welfare: An Evaluation of Global Food Companies’ Discourse and Disclosures on Farm Animal Welfare

**DOI:** 10.3390/ani7030017

**Published:** 2017-03-06

**Authors:** Rory Sullivan, Nicky Amos, Heleen A. van de Weerd

**Affiliations:** 1 Centre for Climate Change Economics and Policy, School of Earth and Environment, University of Leeds, Leeds LS2 9JT, UK; 2 Nicky Amos CSR Services Ltd., Old Broyle Road, Chichester, West Sussex PO19 3PR, UK; nicky@nicky-amos.co.uk; 3 Cerebrus Associates Ltd., The White House, 2 Meadrow, Godalming, Surrey GU7 3HN, UK; heleen@cerebrus.org

**Keywords:** animal welfare, farm animals, global food companies, CSR, risk management

## Abstract

**Simple Summary:**

Companies that produce or sell food products from farm animals can have a major influence on the lives and welfare of these animals. The Business Benchmark on Farm Animal Welfare (BBFAW) conducts an annual evaluation of the farm animal welfare-related disclosures of some of the world’s largest food companies. The programme looks at companies’ published policies and commitments and examines whether these might lead to actions that can improve animal welfare on farms. It also assesses whether companies show leadership in this field. The BBFAW found that, in 2012 and 2013, around 70% of companies acknowledged animal welfare as a business issue, and that, between 2012 and 2013, there was clear evidence of an increased level of disclosure on farm animal welfare awareness in the companies that were assessed. However, only 34% (2012) and 44% (2013) of companies had published comprehensive farm animal welfare policies, suggesting that many companies have yet to report on farm animal welfare as a business issue or disclose their approach to farm animal welfare to stakeholders and society.

**Abstract:**

The views that food companies hold about their responsibilities for animal welfare can strongly influence the lives and welfare of farm animals. If a company’s commitment is translated into action, it can be a major driver of animal welfare. The Business Benchmark on Farm Animal Welfare (BBFAW) is an annual evaluation of farm animal welfare-related practices, reporting and performance of food companies. The framework evaluates how close, based on their disclosures, companies are to best practice in three areas: Management Commitment, Governance & Performance and Leadership & Innovation. The BBFAW analysed information published by 68 (2012) and 70 (2013) of the world’s largest food companies. Around 70% of companies acknowledged animal welfare as a business issue. Between 2012 and 2013, the mean BBFAW score increased significantly by 5% (*p* < 0.001, Wilcoxon Signed-Rank test). However, only 34% (2012) and 44% (2013) of companies published comprehensive animal welfare policies. This increase suggests that global food companies are increasingly aware that farm animal welfare is of interest to their stakeholders, but also that many companies have yet to acknowledge farm animal welfare as a business issue or to demonstrate their approach to farm animal welfare to stakeholders and society.

## 1. Introduction

Nearly 70 billion animals are farmed globally each year for meat, milk and eggs; the share of intensive landless systems (mainly pork and chicken) is about 45% of total meat output [1]. Animal production has intensified for a variety of reasons, in particular increased demand for food and ongoing pressure to reduce the costs of production for producers and food companies [2]. However, modern commercial practices have also raised concerns about farm animal welfare [3]. The underlying causes are a mix of the systems and processes used to manage farm animals (e.g., the type of housing) and of the competence and diligence of the individuals charged with managing these animals. The most important welfare issues are associated with housing conditions, genetic selection and breeding, management methods (e.g., mutilations), transport and slaughter [4,5,6,7,8,9,10,11,12,13,14,15,16,17]. Issues with housing includes the close confinement of pigs (sow stalls, farrowing crates, single penning, tethering, high stocking densities), cattle (feedlots or concentrated animal feeding operations (CAFOs), tethering, veal crates), poultry (conventional non-enriched cages, high stocking densities) [5,6,7,8] and finfish (high stocking densities, solitary close confinement) [9]. Genetic selection and intensive breeding has produced fast-growing farm animals but puts enormous strain on these animals’ skeletal structure and physiology [7], and severe effects on health and welfare and high rates of mortality can be caused by genetic engineering (cloning) techniques [10,11]. Management procedures associated with adverse welfare (pain, distress) that are routinely used in farming systems are applied to pigs (castration, teeth clipping, tail docking), poultry (toe clipping, beak trimming, desnooding, de-winging), cattle (disbudding, dehorning, castration), sheep (mulesing, castration) and finfish (fin clipping) [9,12,13]. Transport and the associated handling during loading and unloading exposes farm animals to multiple stressors (such as hunger, thirst, discomfort, pain, frustration, fear, distress, injury, disease and death), which can negatively affect their welfare [9,14]. As the journey length increases, animals become increasingly hungry, fatigued and dehydrated and the risk of morbidity and mortality increases. Killing farmed animals in a humane way involves pre-slaughter stunning, rendering the animal unconsciousness and insensible to pain, discomfort and stress, until death occurs; the induction of unconsciousness should be non-aversive and should not cause anxiety, pain, distress or suffering [15]. Finally, the over-use of antibiotics has been directly linked to the global increase in antibiotic resistance [16]. This issue is also associated with animal production, as antibiotics are used to improve growth and production (e.g., promote abnormal muscle growth or milk production), often putting excessive strain on the animal’s physiological capabilities. Furthermore, antibiotics are used to prevent infection before it occurs. The need to use antibiotics in this way is exacerbated by the large numbers of animals living in close proximity in intensive farming environments, often in non-hygienic conditions. This can act as a reservoir of resistance with many opportunities for the transfer of drug-resistant bacteria, thereby accelerating spread of resistance [17].

The views that food companies hold about their responsibilities for animal welfare and their management practices and processes have a critical influence on the lives and welfare of farm animals. An individual company’s commitment can be a major driving force to influence the welfare of animals, especially if the commitment expressed is translated into actual behaviour. This is not just a matter of ethical concern. There are compelling business reasons (or ‘business case arguments’) why companies should be concerned about farm animal welfare. These include regulation and legislation, pressure from animal welfare organisations, and brand and market opportunities for companies with higher farm animal welfare standards (see, for example, [2,18,19]). Consumer pressures are important too, with animal welfare exerting an increasingly strong influence on food purchasing decisions [20] (for a challenge to this view, see [21]). However, there is variation in the pace at which businesses involved in animal production have acted to address welfare issues. For example, within the U.S. food industry, retailers have been quicker to react than producers, in large part because of consumer pressure, which has a direct (or potential) impact on their business [22].

Weaknesses in corporate practices and performance on ethical issues such as animal welfare can be a threat to good business performance (e.g., through impacting on reputation, brand, costs); therefore, managing these issues effectively should be an integral part of companies’ risk and cost management processes [18]. Similarly, the potential for these issues to affect costs, revenues, asset values and brand can have knock-on effects on the cost of debt and of equity and so are a subject of interest for investors [2,23,24]. When we look at the reported actions taken by companies—the subject of this paper—we need to recognise that the actions taken will be critically influenced by the ethical views that food companies hold about the welfare of animals and by the pressures (or lack of pressure) on them to adopt high standards of farm animal welfare.

Despite the importance of corporate practices to animal welfare and the business case for corporate action on animal welfare, relatively little is known about how food companies (either individually or as a sector), manage farm animal welfare, for example as part of their corporate social responsibility (CSR) [2]. There may be various reasons for this. The most important one is the limited information provided by companies on their overall farm animal welfare management practices and processes [19]. Another reason is the lack of tools or frameworks that enable a meaningful assessment of individual company performance (in either absolute terms or relative to its industry peers), despite Maloni and Brown providing an expansive framework for the management of social, environmental and ethical (including animal welfare) issues in the food supply chain [2].

This paper addresses some of the gaps in knowledge by presenting the results of the first structured evaluation of the farm animal welfare-related policies, practices, processes, systems, reporting and performance of 70 of the world’s largest food companies. The paper considers whether these companies report that they have established—or are establishing—a management infrastructure (e.g., policies, management accountabilities, objectives and targets) necessary to manage farm animal welfare, and whether they report on the performance outcomes that they are achieving.

The data presented are derived from research conducted by the Business Benchmark on Farm Animal Welfare (BBFAW). The Benchmark is a tool for investors seeking to evaluate the relative performance of food companies on farm animal welfare management. To that end, it assesses company reporting on farm animal welfare using a framework that broadly aligns with the manner in which companies report to investors on other corporate responsibility issues [25] (pp. 14–21); a specific example is the climate change reporting framework developed by the CDP—previously the Carbon Disclosure Project (https://www.cdp.net/en, accessed on 28 October 2016).

BBFAW’s main objective (see Table 1) is to improve farm animal welfare standards in the world’s leading food businesses by providing investors and, albeit to a lesser extent, other stakeholders with an independent, impartial and reliable assessment of food companies’ reported practices and performance. The overarching objective and aims are listed in Table 1, with the underlying assumption that the process of disclosure and the dialogue between investors and companies will stimulate companies’ efforts to adopt higher farm animal welfare standards and practices.

The central deliverable of BBFAW’s work is an annual public benchmark of how global food companies report on how they are managing farm animal welfare. This paper reports on the first two assessments of company performance, the 2012 Benchmark [23] and the 2013 Benchmark [26], and discusses what the results tell us about corporate practices on the reporting on farm animal welfare management. Subsequent Benchmark reports have been produced, but with somewhat different questions and across a significantly extended universe; their data are therefore not included in the current analysis.

## 2. Materials and Methods

The annual BBFAW public benchmark uses information published by global food companies on farm animal welfare to assess how these companies report on how they manage farm animal welfare. This approach is consistent with the idea that companies need to provide sufficient information to enable their stakeholders to hold them to account for their practices and performance. However, there may also be a disconnect between the information reported and actual performance, manifesting itself in two ways. First, companies with poor disclosure but relatively good performance may find themselves penalized; second, companies with good disclosure may receive scores or evaluations that are somewhat better than their underlying performance. We discuss this potential disconnect in Section 4.

### 2.1. Selection of Companies

The overarching objective of the company selection process was to provide a broadly representative sample of the larger (in terms of their turnover and their farm animal footprint) companies active in the European food sector. The primary criterion for selecting these companies was the size of their animal footprint within Europe, with economic significance or turnover used as a crude proxy for this footprint. Specifically, data from Euromonitor’s analysis of the top 50 EU food businesses by sector (www.euromonitor.com/consumer-foodservice) and Deloitte’s annual Global Powers of Retailing report [27] were used to develop a list of the companies to be covered by the Benchmark.

The selected companies were broadly spread across the three food industry subsectors: Food Retailers & Wholesalers, Restaurants & Bars and Food Producers. The coverage included major food companies in most European markets, as well as some North American and Brazilian companies. The companies included in the 2013 Benchmark were broadly the same as those included in 2012, although some adjustments had to be made as a consequence of a large company splitting into smaller ones (‘demerger’). The effect of these changes was an increase in the total number of companies covered by the Benchmark from 68 in 2012, to 70 in 2013.

### 2.2. The Benchmarking Process

The core principle of the benchmarking process was that companies were only assessed on the basis of their published information on farm animal welfare, to encourage better disclosure of information.

The first step in the assessment process was a desktop review of each company’s published information. This involved a detailed review of the material on the company’s corporate (i.e., parent company) websites, the material contained in formal publications such as annual reports, corporate responsibility reports and other publications, the material on subsidiary company websites, and the information provided in press releases, consumer brochures and similar publications.

For each individual company, a summary report that recorded its scores, an explanation for the score awarded and details of the sources of information used for the assessment was prepared. Each company was sent its (confidential) summary spreadsheet with supporting information and given 3–4 weeks to review the information and provide feedback and/or additional information. Company scores were only revised if (a) the company could demonstrate that the assessment had not taken account of information that was in the public domain at the time of the assessment (i.e., credit was not given for information published after the time of the assessment); and/or (b) where the assessor had made an error of interpretation or fact in the assessment. The final individual company reports, showing individual scores and comments for each question, as well as overall company scores and comparable sector scores, were sent (in confidence) to the companies at the time of issuing the final Benchmark report.

The final Benchmark report presented the overall outcome of the benchmarking process with a summary graph indicating in which tier (level of performance in terms of animal welfare) a company was placed. There were six tiers in total, with Tier 1 as the highest level possible, showing leadership in animal welfare.

The same benchmarking process was used for the 2012 and 2013 Benchmarks, with a few exceptions (see below). The assessments were conducted in August and September of each year. A restricted assessment period was used in order to ensure that companies were assessed at broadly the same point in time. The choice of timing was deliberate as the vast majority of firms publish their annual reports and accounts, and their sustainability reports in the first half of the year. That is, by conducting the assessments in August and September, we were able to use the most up-to-date information for most companies. In both years, the assessments were done by the same trained assessors who regularly calibrated their scoring with the framework criteria and also reviewed each other’s assessment reports. This safeguarded consistency in the assessment methodology.

### 2.3. The Assessment Framework

A framework (assessment questions, guidance, scoring) against which all companies were assessed was used. The framework focused on the management systems and processes related to farm animal welfare. The framework was developed by a Technical Working Group comprising experts on food businesses and animal welfare, supported by expert advisors on investment and corporate social responsibility. The draft framework was subsequently subjected to extensive consultation with investors, companies and other stakeholders invited to comment and offer suggestions for improvement (for a detailed description, see [28,29]). The lists with proposed companies for the 2012 and 2013 Benchmarks were also reviewed as part of this consultation process. The consultation was done prior to the framework being used for the 2012 and 2013 benchmarking process and led to some minor changes, primarily the addition of some companies to ensure that relevant comparator companies within particular sub-sectors were included. For more details of the framework development and review process, including details of the organisations that commented on the draft framework, (see [23,26,28,29]).

The assessment framework focused on the management systems and processes that companies should have in place to identify, assess, understand and manage the risks and opportunities associated with farm animal welfare. The primary focus was on the corporate entity (or parent company) as a whole rather than subsidiary companies (or brands), to prevent the ‘filtration effect’ whereby part of the company communication is left behind in the transition from parent company to subsidiary [30]. The Benchmark did consider how companies reported on their management of farm animal welfare issues in specific markets or geographic regions and did give credit for reported innovative practices and processes in these markets and regions.

Company performance was considered in three core areas, as indicated in Table 2. These were (a) Management Commitment & Policy, assessing the company’s policy framework for managing farm animal welfare, including its policies on specific animal welfare issues. Some animal welfare issues are covered by national legislation, however the Benchmark took the approach that companies still needed to have formal policies on these issues, to cover the interests of the animals they have an impact on; (b) Governance & Management, assessing the company’s systems and processes for managing farm animal welfare (responsibilities, objectives and targets, internal controls); (c) Leadership & Innovation, assessing the company’s efforts to advance farm animal welfare more widely.

The core areas were weighted as indicated in Table 2. These initial weightings were chosen to align with similar investor benchmarks and to ensure that the results and subsequent company rankings were not overly sensitive to weightings. They also reflected the fact that reporting on farm animal welfare was relatively immature and so it was considered premature to assign very high weightings to performance on impact. Investors, who were seen as the primary audience for the benchmark, were formally consulted on the weightings and were supportive of the approach adopted [23,28]. Within each of the core areas, each company was evaluated against a number of criteria with scores awarded according to how close the company was to best practice. The number of points awarded for specific criteria within a question corresponded to the level of detail in a company statement. In general, the more detail, the broader the scope and the higher the level of commitment, the more points were awarded (implicit in this is that a higher level of commitment has a potential higher impact on animal welfare). An example is presented in Table 3.

### 2.4. Changes in Methodology: 2013 vs. 2012

The criteria used in the 2012 Benchmark were broadly the same as for the 2013 Benchmark. There were two minor changes. First, one new question was added in the Governance & Management section in 2013, on internal controls (specifically on employee training and on the actions to be taken in the event of non-compliance with the farm animal welfare policy). The effect of this change was to increase the total number of points from 170 to 180 and to increase the total number of points for the Governance & Management section from 75 to 85, representing an increase in the proportion of points for this section from 44% to 47% of the total score (Table 2). Second, the interpretation of the question on whether companies had specific policies on genetically modified animals (GMO) was changed, so that in 2013 companies were required to explicitly state that they would not use genetically modified animals as opposed to more general corporate commitments on the avoidance of genetically modified organisms (e.g., in feed), as was the case in 2012.

### 2.5. Statistical Analysis

In order to test if the increase in overall Benchmark score from 2012 to 2013 was a significant change, a Wilcoxon Signed-Rank test (SPSS v. 22) was performed. The data used for this test were the scores for 68 companies that were assessed in both 2012 and 2013 (excluding the new demerged companies that were added in 2013). The absolute (not the relative) values of the Benchmark scores were analysed, but the scores for 2013 were corrected by deducting the points that (some) companies obtained for the new question on internal controls that was added in 2013, so that a like-for-like comparison could be made between the years. The Wilcoxon Signed-Rank test analysed 55 samples, after removing 13 tied scores (as tied scores cannot be ranked).

## 3. Results

**Figure 1:**
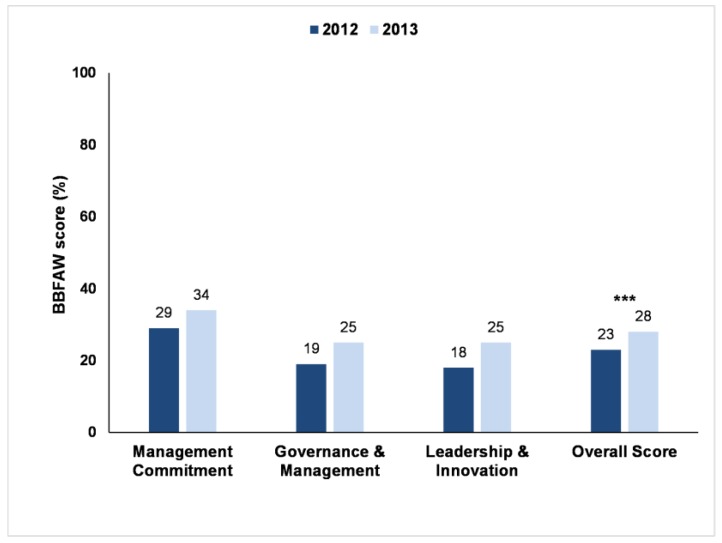
Business Benchmark on Farm Animal Welfare scores (as % of the total possible points) of 68 companies in 2012 and 70 companies in 2013. Average company scores for three core areas of the assessment and average overall company scores in both years. Statistical significance is indicated with *** (*p* < 0.001).

Approximately 70% of the companies assessed in the two Benchmark years acknowledged farm animal welfare as a business issue (71% in 2012, 70% in 2013). Figure 1 shows a comparison of the results of the 2012 and 2013 Benchmarks and illustrates that companies are increasingly reporting that they pay attention to farm animal welfare. The overall benchmark score (the mean of all the overall company scores) increased from 23% in 2012 to 28% in 2013. The Wilcoxon Signed-Rank test showed that this increase in the overall scores was significant (Wilcoxon Signed-Rank test, one-tailed *p* < 0.001). Over this period, the proportion of companies with a published farm animal welfare policy (within the core area ‘Management Commitment’) increased, as did the proportion of companies with published objectives and targets for farm animal welfare.

At the individual company level, companies that achieved an overall Benchmark score of less than 26% were classified in Tiers 5 and 6 (the bottom tiers). These companies provided no evidence that they recognised farm animal welfare as a business issue (Tier 6) or they provided very limited evidence (and limited information on implementation) that this subject was on the business agenda (Tier 5), let alone reporting that they were taking action to address the business risks and opportunities presented by farm animal welfare. In 2013, 53% of the companies were classified in Tier 5 and 6; this figure was higher in 2012 (62% of companies in Tier 5 or 6), showing a slight improvement in overall ranking.

At the other end of the spectrum, companies that achieved an overall Benchmark score of more than 62% were classified in Tiers 1 and 2. Tier 1 companies were those that were considered to be showing leadership through having strongly stated commitments to animal welfare and detailed reporting on how these were being implemented [23,26,28,29]. Tier 2 companies were those that were considered to have animal welfare as an integral part of their reported business strategies, with well-developed (published) management systems and processes and a clear focus on farm animal welfare outcomes. In 2013, 10% of companies were placed in Tiers 1 and 2 (seven companies in total). This was an increase in comparison to the 2012 Benchmark, when only 4% of companies (three companies) were assessed to have this highest level of performance. While there was a general increase in total scores (44 companies) and movement of companies towards higher tiers (11 companies jumped up one tier and eight companies jumped up two tiers in 2013), there were also five companies that dropped by at least one tier and 13 companies that did not change scores. In most cases a fall in tier level appeared to have been caused by changes in reporting (e.g., revamping of corporate websites and, in the process, removing relevant information that was previously published), rather than changes in published policies and practices.

The addition of the new question on internal controls to the Governance & Management section in 2013 had no effect on the tier ranking of companies. While some companies saw a modest (typically less than 1%) increase or decrease in their percentage scores, none saw their overall tier rankings increase or decrease.

### 3.1. Overarching Farm Animal Welfare Policies

**Figure 2:**
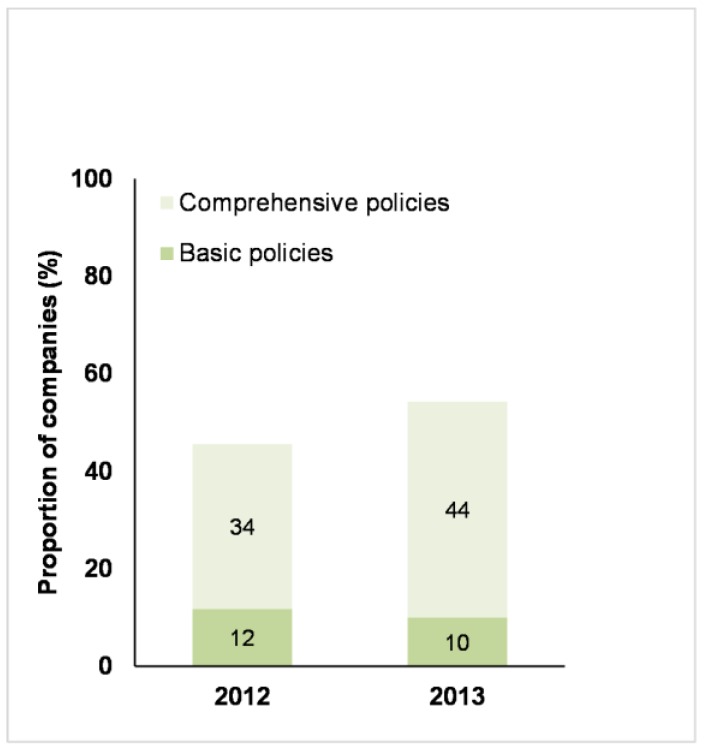
Business Benchmark on Farm Animal Welfare: the proportion of 68 companies in 2012 and 70 companies in 2013 that published either basic or comprehensive policies on farm animal welfare.

The Benchmark differentiated between companies that were considered to have a basic farm animal welfare policy (broadly defined as having a clear published statement of commitment to farm animal welfare and/or farm animal welfare-related principles, but providing limited information on how the policy was to be implemented) and those that published a comprehensive farm animal welfare policy. In order to be considered comprehensive, a farm animal welfare policy needed to include most or all of the following elements: a clear statement of the reasons why farm animal welfare is important to the business; a commitment to compliance with relevant legislation; a clear position with regard to expected standards of farm animal welfare; a description of the processes in place to ensure that the policy is effectively implemented; and a commitment to public reporting on performance [28,29]. The number of companies that had published (any) farm policies, increased from 2012 to 2013 (Figure 2), mainly due to an increase in companies publishing comprehensive farm animal welfare policies. Of the companies that had published comprehensive or basic farm animal welfare policies, 77% and 79% (for 2012 and 2013, respectively) applied these policies to all geographies; 58% and 68% (for 2012 and 2013, respectively) applied these policies to all relevant animal species and 48% and 45% (for 2012 and 2013, respectively) applied these policies to all products produced, manufactured or sold.

### 3.2. Policies on Specific Farm Animal Welfare Issues

**Figure 3:**
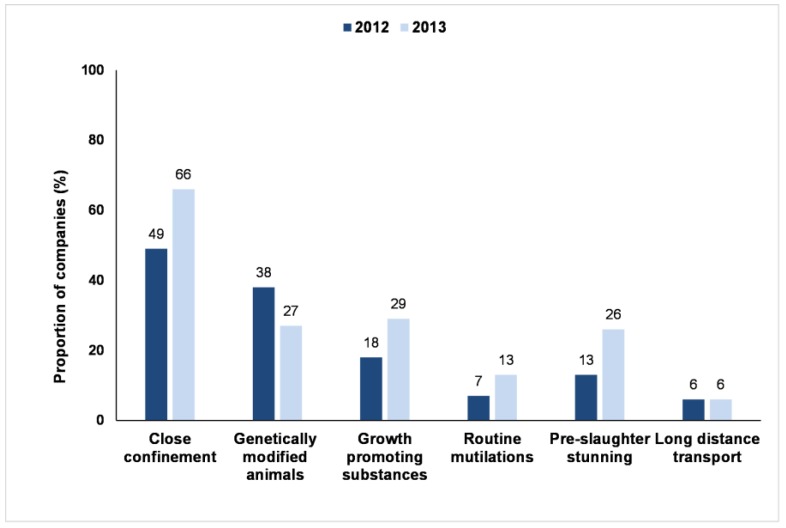
Business Benchmark on Farm Animal Welfare: the proportion of 68 companies in 2012 and 70 companies in 2013 with specific policies on six key farm animal welfare-related issues.

Figure 3 indicates the proportion of companies that made at least partial commitments to six key farm animal welfare-related issues included in the Benchmark. Between 2012 and 2013, there was an increase in the proportion of companies with published policies on each of these issues, with the exception of long-distance transport (not changed) and Genetically Modified Organisms (GMOs), which showed a decrease. The change in the proportion of companies considered to have GMO policies related to the change in methodology, discussed in the methods section, on how this question was interpreted between 2012 and 2013.

Overall, few companies published specific policies on animal welfare issues, the exception being policies on close confinement. Relatively few companies made commitments to the complete avoidance of various welfare practices; most only made partial commitments. Policies were, generally limited to particular species, geographies or product segments. For example, for routine mutilations, of the 13% of companies with a policy on this issue in 2013, 4% were unclear about the scope of their commitment, 6% limited the scope to particular geographic regions, species or products, and just 3% had made a universal commitment to the avoidance of routine mutilations.

### 3.3. Governance and Management

More than half of the companies (59% in 2012; 54% in 2013) assessed did not provide any information on who was responsible for farm animal welfare, at either a senior management or operational level.

**Figure 4:**
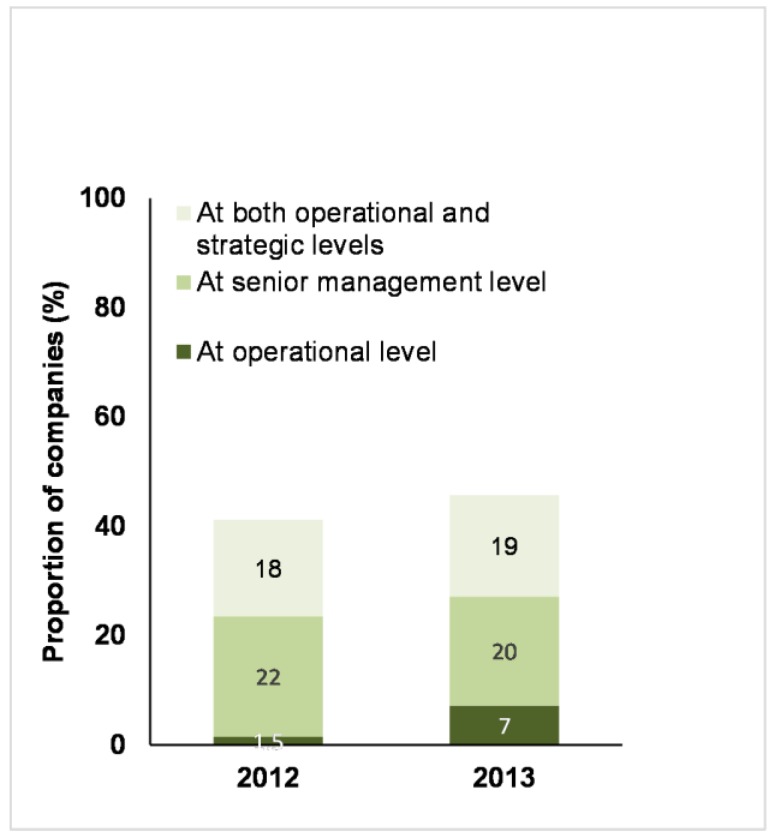
Business Benchmark on Farm Animal Welfare: the proportion of 68 companies in 2012 and 70 companies in 2013 that published who was responsible for managing farm animal welfare and at which level.

Around 40% of companies provided this information in 2012 and this percentage increased in 2013 (Figure 4). These companies specified who (i.e., the individual or the position) had operational responsibility for farm animal welfare, who at senior management or board level had oversight responsibility for farm animal welfare, or provided information on both operational and strategic responsibilities (Figure 4).

### 3.4. Objectives and Targets

**Figure 5:**
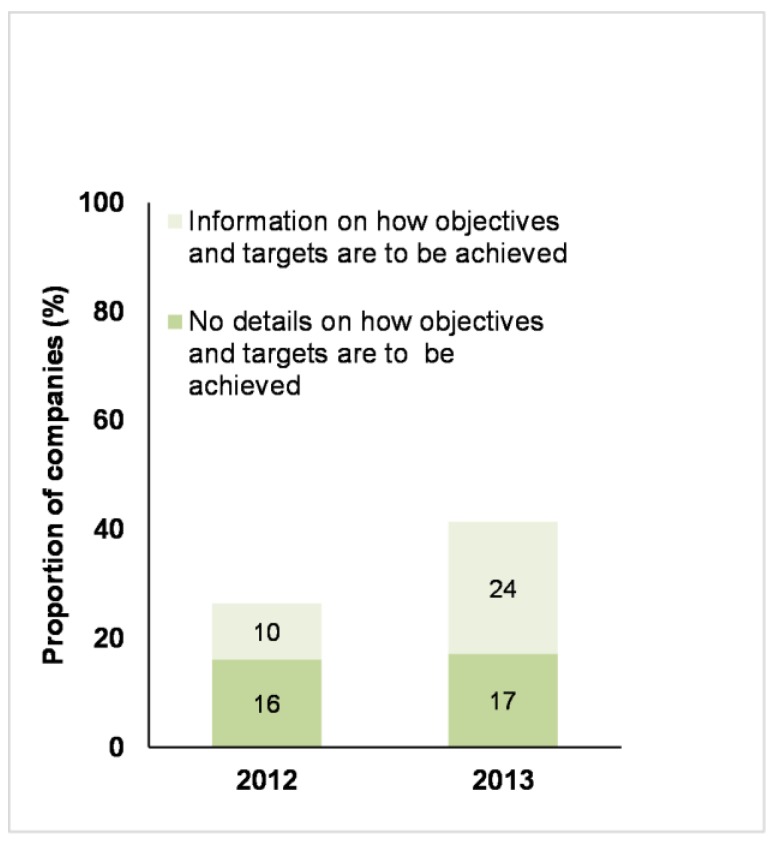
Business Benchmark on Farm Animal Welfare: the proportion of 68 companies in 2012 and 70 companies in 2013 that published objectives and targets for their farm animal welfare policy, with or without details on how to achieve these objectives.

In 2013 there was an increase in the number of companies that reported that they had set farm animal welfare-related objectives and targets (Figure 5). There was also an increase in the number of these companies that provided a reasonable amount of information on how the targets were to be achieved (for example, who was responsible, what resources were allocated, what were the key steps or actions towards the target) (see Figure 5).

### 3.5. Supply Chain Management

**Figure 6:**
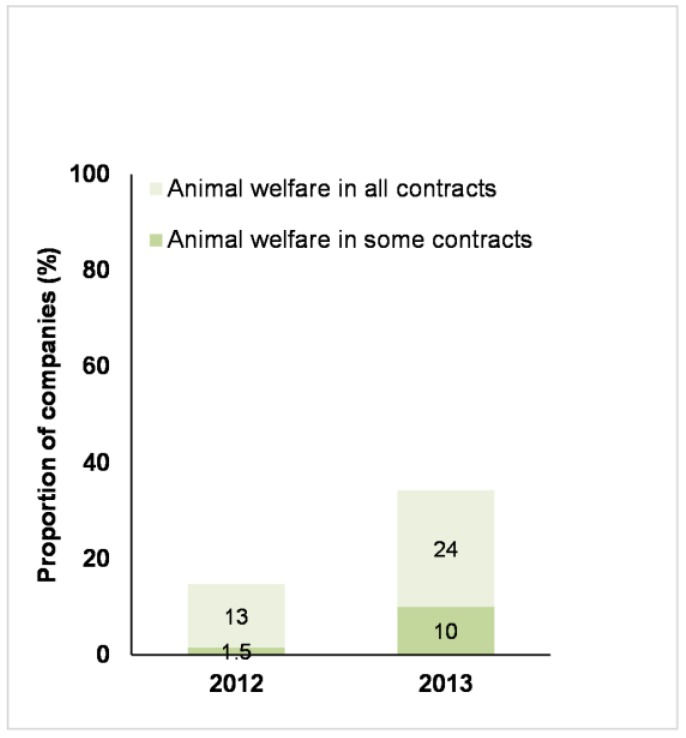
Business Benchmark on Farm Animal Welfare: the proportion of 68 companies in 2012 and 70 companies in 2013 had farm animal welfare in all or some of their supplier’s contracts.

The number of companies discussing how farm animal welfare was included in supplier contract conditions increased between 2012 and 2013 (Figure 6). The majority of these companies reported that they included farm animal welfare in all relevant contracts, suggesting they have a comprehensive approach to farm animal welfare in their supply chains. The other companies reported that they included farm animal welfare in some contracts but did not specify the proportion of contracts where farm animal welfare was included.

While companies increased the amount of information they provided on their supply chain management processes, most provided limited information on the actual standards of farm animal welfare in their supply chains. For example, in 2013, only 43% of the companies (35% in 2012) described how they audited their suppliers, and only 34% described their supplier education and capacity-building initiatives (31% in 2012).

### 3.6. Reporting on Farm Animal Welfare Performance

**Figure 7:**
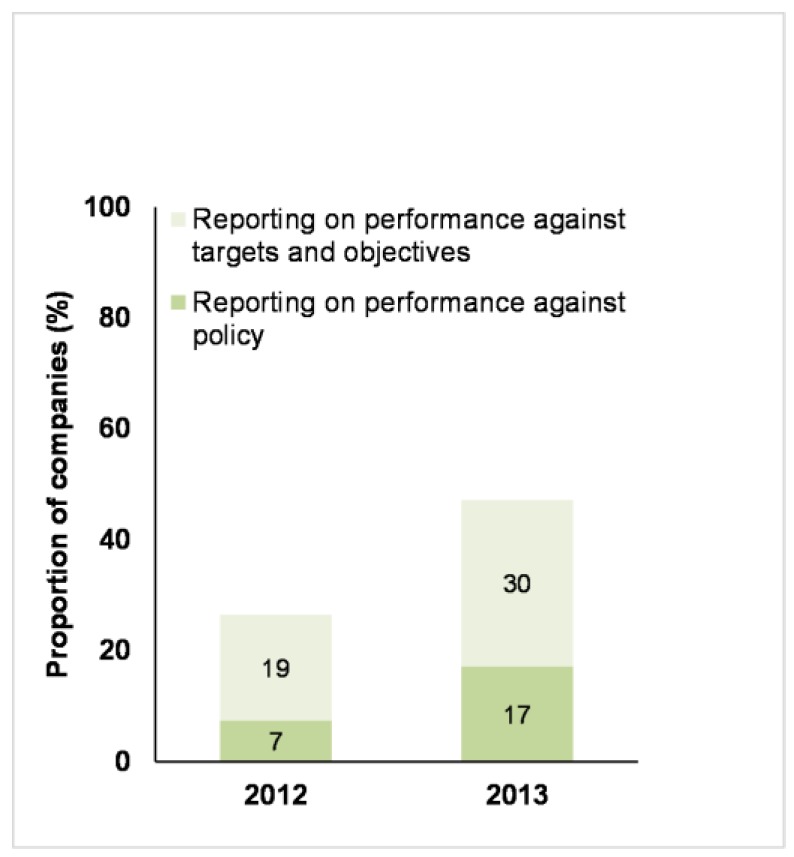
Business Benchmark on Farm Animal Welfare: the proportion of 68 companies in 2012 and 70 companies in 2013 that reported on their performance against their farm animal welfare policy or targets and objectives.

Performance reporting by companies remains relatively underdeveloped. In the 2013 Benchmark, only 17% of the 70 companies reported on how they performed against their policy commitments, and 30% reported on their performance against their objectives and targets. However, these numbers did represent increases from the 2012 Benchmark (Figure 7).

### 3.7. Assurance Schemes

**Figure 8:**
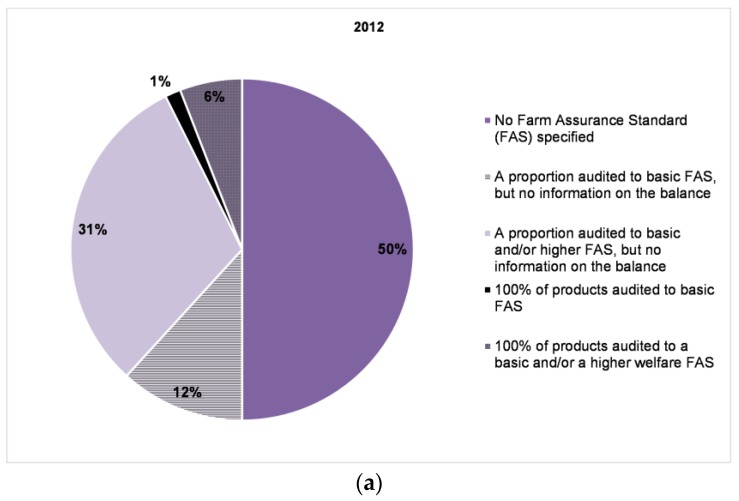

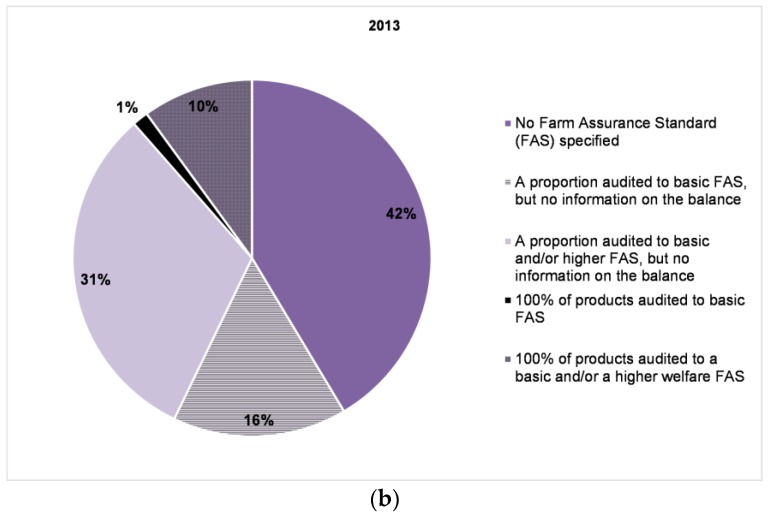
Business Benchmark on Farm Animal Welfare: (**a**) proportion of 68 companies in 2012 and (**b**) proportion of 70 companies in 2013 that reported on the farm assurance schemes, FAS (or standards) to which their animals were reared, transported and slaughtered. None of the companies (in either year) had 100% of their products audited to higher level welfare farm assurance standards.

About half of the companies (50% in 2012; 60% in 2013) assessed by the Benchmark provided at least some information on the assurance schemes (or standards) to which their animals were reared, transported and slaughtered (Figure 8). None of the companies, in either 2012 or 2013, had all of their products audited to higher level assurance standards (e.g., NL Beter Leven 3*). The largest proportion were companies stating that a proportion of their products or farms were audited to a basic farm assurance standard (e.g., UK Red Tractor), but they provided no information on the balance.

### 3.8. Promoting Farm Animal Welfare

**Figure 9:**
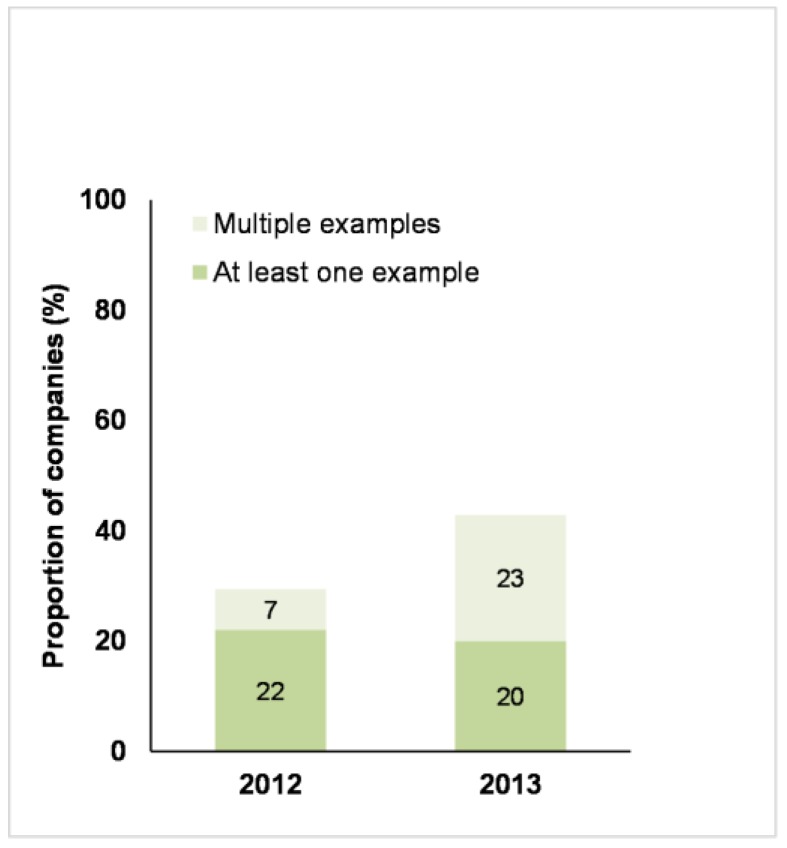
Business Benchmark on Farm Animal Welfare: the proportion of 68 companies in 2012 and 70 companies in 2013 that provided information (either at least one example or multiple examples) to their customers or consumers on farm animal welfare.

Figure 9 shows that there was an increase in the number of companies that provided information to their customers or consumers on farm animal welfare. The number of companies that presented multiple examples increased in 2013.

### 3.9. Sectoral Analysis

**Figure 10:**
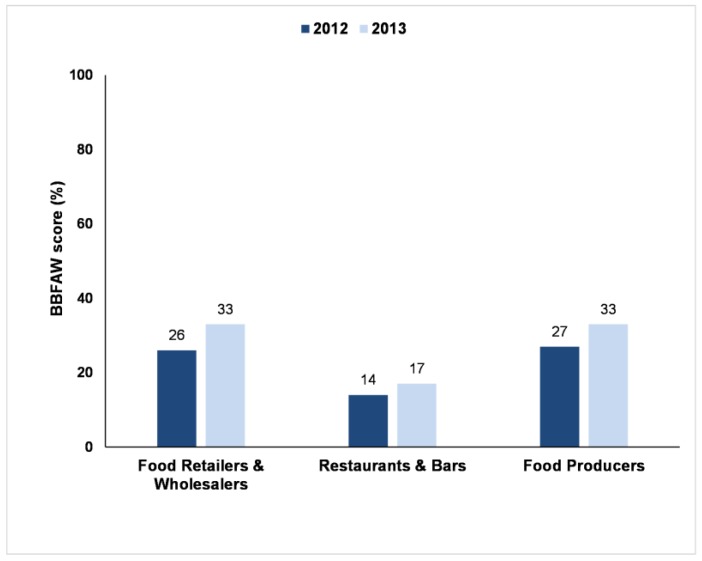
Business Benchmark on Farm Animal Welfare scores (as % of the total possible points) of 68 companies in 2012 and 70 companies in 2013. Average scores for the three sub-sectors (company types) in each year.

Figure 10 presents the results broken down for each sub-sector. In comparison to the 2012 Benchmark, the overall average scores for both Food Retailers & Wholesalers and for Food Producers had increased by 7%, but the average score for Restaurants & Bars had only increased by 2%.

### 3.10. Company Feedback

Companies were given the opportunity to comment on the confidential initial results reports they received. In 2012, approximately half of the companies covered by the Benchmark provided additional information, compared to approximately one-third in the 2013 iteration. A number of individual company scores were revised based on the information provided, as per the Benchmark criteria for revising scores (see Materials and Methods).

## 4. Discussion

The overall score (the mean of the scores across the three core areas: Management Commitment, Governance & Management, and Leadership & Innovation) for the 2013 Benchmark had increased by 5% in comparison with 2012, which was a significant change. This was caused by 44 companies (out of 68 companies) increasing their total Benchmark score (and 19 companies actually moving up at least one tier) and shows that companies were increasingly reporting on the attention they pay to farm animal welfare.

The overall findings are similar to findings presented by Janssens and Kaptein, who analysed the websites of the 200 largest corporations in the world (selected from the 2012 Fortune Global 500 list) for statements of responsibility towards animals [19]. Their assessment of 21 companies involved in animal-based food products concluded that 76% of these companies had made statements of responsibility towards animals, a broadly similar figure to the 71% (2012) and 70% (2013) of the companies covered by the Benchmark who acknowledged farm animal welfare as a business issue.

The 2013 Benchmark shows that companies are increasingly reporting on their management infrastructure (starting with policies, then management systems and processes, and then performance reporting) to ensure that they manage farm animal welfare effectively. There could be various reasons why companies are doing this. One likely reason is pressure on companies from stakeholders to effectively manage farm animal welfare-related issues. This pressure comes from stakeholders who see ethical animal issues as part of business ethics, global consumers who believe it is important to protect the welfare of farmed animals and who are interested in buying higher welfare products [21,31,32], animal welfare NGOs and, regulation, in particular within the EU [33].

Companies have sought to reassure consumers by publishing more information on the management of their supply chains, covering issues such as monitoring, testing, supplier training and auditing. They have also sought to reassure their investors that they are effectively managing the risks related to food provenance, traceability and quality. Many companies have concluded that ignoring supply chain-related issues may create business risks, with many deciding that it is in their financial best interests to proactively prepare a comprehensive strategy for managing supply chain CSR [2].

Despite the positive trends in corporate disclosures, less than half of the companies included in the 2013 Benchmark (46%) had published comprehensive farm animal welfare policies, and another 10% had only published a basic policy statement. While this shows progress in comparison with the 2012 Benchmark where just 34% of companies had published comprehensive policies (and 12% had basic policy statements), it is important to recognise that many of these policies were quite limited in terms of their scope and there was only limited information on how the policy was to be implemented. The existence of a policy does not guarantee implementation in western multinationals (see discussion on this topic below), however, the absence of a policy is often interpreted by investors as a signal that the issue in question is not on the corporate agenda [23,25] (pp. 70–72) [34] (p. 17). This suggests that farm animal welfare is not seen as a corporate priority by many companies, and that companies may not have the management infrastructure necessary to ensure that these policies are implemented effectively. The implication, supported by the data presented in this article, is that many companies are not monitoring or managing farm animal welfare in their supply chains and this increases the risk that the welfare of farm animals is not being properly monitored and managed. It may also mean that companies will be slow to act in situations where animal welfare is being compromised or that the actions taken may be ineffective.

The slightly higher average company scores for Management Commitment compared to Governance & Management and Leadership & Innovation suggest that companies’ awareness of the importance of farm animal welfare is growing and that they are starting to develop the policy frameworks needed to effectively manage these issues. Despite this, progress is variable, as many of the published overarching policies on farm animal welfare have limitations in terms of the geographic regions they apply to, the species and/or the products covered (e.g., own-brand vs. all products).

More than half of the companies assessed (2012: 59%, 2013: 54%) did not provide any information on who is responsible for farm animal welfare, at either a senior management or operational level. Furthermore, it was frequently difficult to tell how much, if any, senior management attention was actually focused explicitly on farm animal welfare. In most cases, farm animal welfare was presented as just one of a whole range of corporate responsibility-related issues that were reportedly overseen by senior management.

Within the group of companies with overarching farm animal welfare policies, only a few also reported to have established formal policies on specific farm animal welfare issues (such as on mutilations, transport duration etc.). This reflects the normal evolution of corporate responsibility practice—as has been seen in areas such as climate change [35,36]—where companies tend to start with high level policies and, over time, as they gain greater knowledge of the issue in question, formulate more detailed policies on specific issues.

A number of the companies that provided feedback on the 2012 and 2013 Benchmark results argued that the fact that some of these issues are covered by legislation removed the need for them to have a formal policy on it. A commonly cited example is the requirement for pre-slaughter stunning (with a few exemptions), that is currently part of EU law. However, one of the limitations of legislation is that it is rarely comprehensive across all species (either in terms of the issues covered or geographic scope), and its effectiveness is dependent on the level of enforcement [33]. This is particularly important when considering the complexity of supply chains where product ingredients can be sourced from a variety of jurisdictions. Therefore, the approach adopted in the Benchmark was to require companies to have public formal policies that applied to their own operations and their suppliers, and covered the interests of the animals they have an impact on.

In line with the general trends in improvements across all aspects of the Benchmark in this period, the 2013 Benchmark showed an increase in the number of companies that published formal public policy commitments on specific animal welfare issues, except for long distance transport (no change) and on the use of genetically modified animals (decrease). However, this was primarily caused by the change in the Benchmark methodology (as the focus of this question was narrowed to require companies to explicitly state that they would not use genetically modified animals as opposed to more general public corporate commitments on the avoidance of genetically modified organisms, e.g., in feed).

One specific farm animal welfare issue, close confinement, was an exception in terms of companies having a formal public policy on it. Almost 50% (2012) and 66% (2013) of companies published a specific policy on this issue. Most policies related, depending on the company, to the sourcing of cage-free eggs or to the sourcing of pig meat from sows who had not been constrained in sow (gestation) stalls and/or farrowing crates. It is likely that this trend has been driven by a range of factors, notably public pressure, NGO campaigns and changes in legislation in some parts of the world (e.g., the EU Pig Council Directive 2008/120/EC, which lays down minimum standards for the protection of pigs [37]; see also [38] (pp. 101–115)). In some cases companies have made formal, public commitments to the elimination of such practices, even in the absence of an overarching farm animal welfare policy.

With regards to farm animal welfare-related objectives and targets, the increase in the number of companies with published specific objectives and targets was encouraging. In most cases, the targets reflected the relative novelty of farm animal welfare as a management issue, with companies tending to focus on management processes (for example, to formalise their farm animal welfare management systems, to introduce audits) and/or on a single farm animal welfare-related issue (for example, to eliminate the use of gestation crates, or to move towards cage-free eggs).

The Benchmark indicates that reporting on farm animal welfare, although improving, remains relatively underdeveloped. In addition, companies regularly put their actions or results in documents of marginal importance or ‘hidden’ documents of a low status or with a temporary nature (e.g., company magazines or blogs) rather than in online corporate-level documents. This limits communication about such actions or outcomes [19]. There may be various reasons for this. Companies generally have multiple animal species in their supply chain that are frequently managed to different standards which makes it difficult to reduce performance to a single data point. Furthermore, companies may be concerned that reporting on performance will lead to them being criticised, especially if other companies do not provide equivalent levels of disclosure, or if disclosure is impeded due to commercial confidentiality [39].

It was reassuring to find that 60% of the companies covered by the 2013 Benchmark provided at least some information on the assurance schemes (or standards) to which their animals are reared, transported and slaughtered. Assurance schemes can play an important role in promoting and applying farm animal welfare standards [40]. Membership of assurance schemes and the associated inspections of livestock holdings can lead to a reduced risk of non-compliance with legislation and reduced unnecessary pain or distress present on farm [41]. Assurance schemes also provide many of the core process elements (e.g., on auditing, on traceability) that companies need if they are to implement effective farm animal welfare management processes in their supply chains.

The number of companies that provided information on farm animal welfare to their stakeholders (e.g., customers and investors) almost doubled in 2013 (43%) compared to 2012 (25%). This suggests that farm animal welfare has become a more integral part of customer messaging and engagement, rather than a one-off initiative. This may be a consequence of the public Benchmark assessments, putting pressure on companies to strengthen their disclosures on farm animal welfare. This should be a win-win situation, as a company that communicates what it stands for and how it performs, makes it easier for customers and other stakeholders to hold them to account and also stimulates the company to fulfil its responsibilities [42,43].

The Benchmark results for each sub-sector (i.e., Food Retailers & Wholesalers, Restaurants & Bars and Food Producers) show that performance across all three of the sectors is relatively poor. Furthermore, the Restaurants & Bar sector continues to be a noticeably poorer performer than the other two sectors and the gap with these sectors widened further in 2013, compared to 2012. The sub-sectors also show a different degree of change, the Restaurants & Bars sector only marginally increasing their overall score. The reasons for this variation in performance are unclear, but it may be because not all companies in the Restaurants & Bars sector are highly visible to the general public. For example, the sub-set of companies in the Restaurants & Bars sector that had a strong high street presence and who traded under their corporate brand name, had an average score of 27%, which was broadly similar to the average score for the other two sub-sectors (Food Retailers & Wholesalers and Food Producers).

One of the main conclusions from the 2012 and 2013 Business Benchmarks on Farm Animal Welfare is that, despite signs of progress, reporting of corporate practice on farm animal welfare lags behind practice of reporting on other corporate responsibility issues (see, for example, data on climate change in [35,36]). This may, in part, reflect the focus on published information in the Benchmark methodology, as many companies have argued that they do much more behind the scenes, but that they do not always report on these activities. Even if this were true, the lack of disclosure (and the consequent low overall Benchmark scores) suggests that companies are not well prepared to report on their performance. Businesses that refrain from publishing policy documents that are coupled to actual behaviour and operations aligned to the goals of that policy will increasingly be confronted with stakeholders who want to know why a policy is not viewed as a desirable instrument to manage ethics, integrity and social responsibility [42,44].

An important point of discussion is whether the Benchmark affects the quality of life of farm animals, as that is its ultimate aim. There may be a lack of connection between companies’ disclosures and their actual performance (see for a comparable issue on CSR policy disclosure: [45]). It is possible that some companies scored well in the Benchmark, simply because they have good disclosure, instead of actually achieving good performance outcomes. That is, there may be a structural disconnect (or decoupling) between organisational policies and organisational practices [44]. Addressing this disconnect requires ethics programmes to be truly integrated into other performance management processes, such as performance monitoring systems tracking (un)ethical behaviour, training programs integrating instruction on ethical processes, and reward/punishment systems attending not only to business results, but also on how those results were achieved [44]. Another possibility is that some companies are doing an excellent job of managing farm animal welfare but score poorly because their disclosure is not sufficiently detailed or robust, or document location is inadequate (e.g., not at corporate level). One of the priorities for future iterations of the Benchmark is to progressively introduce performance criteria, with a greater focus on performance outcomes, into the Benchmark. This will include reporting on both input-based and outcome-based measures [39]. Input-based measures focus on housing and husbandry provisions, such as the type of production system (e.g., cage, barn, free-range), relevant aspects of housing (e.g., space allowance, provision of environmental enrichment), treatments and procedures, breed use, feeding and health management (e.g., the use of preventative antibiotics), and transport and slaughter practices. Outcome-based measures focus on an animal’s current welfare state, while integrating long-term consequences of past husbandry [46]. These measures are specific to individual species, e.g., lameness and mastitis in dairy cows, gait score and footpad dermatitis in broilers, tail-biting and lameness in pigs, and bone breakage and feather cover in laying hens. Outcome-based measures are not confined to physical measures of wellbeing but also include aspects of mental wellbeing (e.g., reaction to humans or novelty, fear, comfort) and behaviour (e.g., time spent lying down, resting or ruminating; or being active, foraging, perching, dust-bathing or socialising). Relevant outcome- and input-based measures correlate with increased welfare [46].

The Business Benchmark on Farm Animal Welfare will be repeated on an annual basis, with the plan being to increase its coverage to 100 global food companies and to increase its focus on animal welfare performance (reporting on outcome measures). This reflects the trend in responsible investment to move beyond a process-focused approach of management systems and reporting, to focus more on the actual social and environmental performance of companies [25]. A focus on performance should allow for simultaneous tracking and influencing of corporate practices on farm animal welfare and, over time, contribute to meaningful improvements in welfare that make a real difference to farm animals.

## 5. Conclusions

The findings from the 2012 and 2013 Business Benchmarks on Farm Animal Welfare suggest that many of the world’s largest food companies have yet to formally and publicly acknowledge farm animal welfare as a business issue. Furthermore, many companies have yet to establish robust farm animal welfare management systems and processes, and many have yet to provide a comprehensive account to their stakeholders and to wider society of their approach to farm animal welfare. Corporate practice and reporting on farm animal welfare remains relatively underdeveloped, but there are encouraging signs of progress. One of the reasons for this may be the contribution of the Business Benchmark on Farm Animal Welfare. While it is premature to offer a definitive assessment, there are signs that the Benchmark is driving change by enabling companies to benchmark themselves against their industry peers, and by providing companies with a clear set of expectations [47].

It may be too early to see the impact of the BBFAW on animals’ lives, but there are clear signs of an increase in the attention being paid to animal welfare in company disclosures, as illustrated by the significant increase in scores that companies achieved in the first two years of Benchmarking discussed in this paper.

Ultimately, the views that food companies hold about the welfare of animals in their realm of influence and their management practices and processes are of critical importance in determining the welfare of billions of farm animals.

## Figures and Tables

**Table 1:** The overall objective and three aims of the Business Benchmark on Farm Animal Welfare.

Objective	To Drive Higher Farm Animal Welfare Standards in the World’s Leading Food Businesses
Aim 1	To provide investors with the information they need to understand the business implications of farm animal welfare for the companies in which they are invested
Aim 2	To provide investors, governments, academics, NGOs, consumers and other stakeholders with an independent, impartial and reliable assessment of individual company efforts to adopt higher farm animal welfare standards and practices
Aim 3	To provide guidance * to companies interested in improving their management and reporting on farm animal welfare issues

* BBFAW produces a range of materials on issues such as the business case for farm animal welfare, best practices in management and reporting, and new/forthcoming farm animal welfare-related regulations and policies. Furthermore, BBFAW conducts structured and extensive engagement programmes, encouraging investors to pay more attention to farm animal welfare in their investment processes and companies to improve their practices, performance and reporting on farm animal welfare.

**Table 2:** Business Benchmark on Farm Animal Welfare: core areas and key elements of the scoring framework.

Core Area and Key Elements	No. of Points	Weighting (% of Total Score)
1. Management Commitment:	65	36%
General account of why farm animal welfare is important to the business, including discussion on the risks and business opportunities. Overarching farm animal welfare policy that sets out core principles and beliefs on farm animal welfare and that explains how these are addressed and implemented throughout the business. Specific policy positions on key welfare concerns such as the close confinement of livestock, animals subjected to genetic engineering or cloning, routine mutilations, slaughter without stunning, and long distance live transportation.		
2. Governance & Management:	85 (75 in 2012)	47% (44% in 2012)
Defined responsibilities for the day-to-day management of animal welfare-related issues as well as strategic oversight of how the company’s policy is being implemented. Objectives and targets including process and performance measures (with an explanation of how they are delivered and how progress is monitored). Outcomes in terms of performance against objectives and targets, performance against company policy and animal welfare outcomes. Internal controls such as employee training in farm animal welfare and the actions to be taken in the event of non-compliance with the farm animal welfare policy. Policy implementation through supply chains, including formalising farm animal welfare in supplier contracts, supply chain monitoring and auditing processes, and supporting suppliers in meeting the company’s standards on farm animal welfare.		
3. Leadership & Innovation:	30	17%
Company involvement in research and development programmes to advance farm animal welfare. Company involvement in industry or other initiatives directed at improving farm animal welfare. Acknowledgement of farm animal welfare performance from notable award or accreditation schemes. Company initiatives to promote higher farm animal welfare amongst customers or consumers.		
**Total**	180 (170 in 2012)	100%

**Table 3:** Business Benchmark on Farm Animal Welfare: sample scoring of an individual question.

Does the company have a clear position on the avoidance of close confinement for livestock (i.e., no sow stalls, concentrated animal feeding operations (CAFOs), feedlots, farrowing crates, single penning, battery cages, tethering or veal crates)?	
No stated position	Score: 0 points
The company has made a partial commitment to the avoidance of confinement but the scope (in terms of geography, species, products) is not clearly defined.	Score: 1 point
The company has made a partial commitment to the avoidance of confinement and the scope of the commitment (in terms of geography, species, and products) is clearly defined.	Score: 3 points
Universal commitment to avoid confinement across all relevant species, own-brand products and geographies.	Score: 5 points
**Maximum Score:**	**5 points**
